# Edentulism and prosthetic rehabilitation needs in the european adult population: A health survey analysis

**DOI:** 10.1590/0103-644020266851

**Published:** 2026-07-24

**Authors:** Diego Gómez-Costa, Rocío Cascos Sánchez, José Luis Antonaya Martín, Noelia Rivas Martín, Pablo Lastra Prados

**Affiliations:** 1 Master's Programme in Implant-Supported Prostheses, Universidad Rey Juan Carlos - URJC Spain; 2 Lecturer in the Master's Programme in Implant-Supported Prostheses, Universidad Rey Juan Carlos - URJCSpain

**Keywords:** Dental Public Health, Edentulism, Epidemiology, Health Surveys, Dental Prosthesis, Dental Implants

## Abstract

To analyse the prevalence of complete and partial edentulism in the European adult population and to evaluate demographic and geographical factors associated with tooth loss and prosthetic rehabilitation needs using national health survey data. A cross-sectional ecological study used aggregated secondary data from the European Health Interview Survey (EHIS), Eurobarometer, and national oral health surveys from Germany, Spain, France, Italy, Poland, Romania, Sweden, and the United Kingdom. Surveys published between 2010 and 2023 were included, representing more than 250,000 adults. Prevalence of complete and partial edentulism was analyzed by age group, sex, and geographical region. Descriptive statistics were calculated as percentages and absolute numbers with 95% confidence intervals. A generalized linear model with binomial distribution estimated adjusted odds ratios for complete edentulism. Statistical significance was set at α = 0.05. Complete edentulism among adults aged 65-74 years ranged from less than 6% in Northern and Western Europe to more than 40% in Eastern Europe, corresponding to approximately 3,200 to over 4,500 individuals. Partial edentulism exceeded 65% in adults aged 45 years and older across all regions. Multivariable analysis identified advanced age as the strongest predictor of complete edentulism (aOR = 28.4 for ≥75 vs. 45-54; p < 0.001), followed by residence in Eastern Europe (aOR = 9.8; p < 0.001). Sex was not independently associated with complete edentulism. Edentulism prevalence in Europe varies by age and geographical region. These findings support region-specific planning of prosthetic rehabilitation services and public health strategies to reduce oral health inequalities.

## Resumo

Analisar a prevalência do edentulismo total e parcial na população adulta europeia e avaliar os fatores demográficos e geográficos associados à perda dentária e às necessidades de reabilitação protética, utilizando dados de inquéritos nacionais de saúde. Este estudo ecológico transversal utilizou dados secundários agregados do European Health Interview Survey (EHIS), do Eurobarômetro e de inquéritos nacionais de saúde bucal da Alemanha, Espanha, França, Itália, Polônia, Romênia, Suécia e Reino Unido. Foram incluídos inquéritos publicados entre 2010 e 2023, representando mais de 250.000 adultos. A prevalência do edentulismo total e parcial foi analisada por faixa etária, sexo e região geográfica. As estatísticas descritivas foram calculadas em porcentagens e números absolutos com intervalos de confiança de 95%. Modelo linear generalizado com distribuição binomial estimou as razões de chance ajustadas (aOR) para o edentulismo total. O nível de significância estatística foi definido em α=0,05. Resultados: O edentulismo total entre adultos de 65 a 74 anos variou de menos de 6% no norte e oeste da Europa a mais de 40% no leste europeu, correspondendo a aproximadamente 3.200 a mais de 4.500 indivíduos. O edentulismo parcial superou 65% em adultos com 45 anos ou mais em todas as regiões. A análise multivariada identificou a idade avançada como o preditor mais forte para o edentulismo total (aOR=28,4 para ≥75 vs. 45-54 anos; p<0,001), seguido pela residência no leste da Europa (aOR=9,8; p<0,001). O sexo não apresentou associação independente com o edentulismo total. A prevalência do edentulismo na Europa varia de acordo com a idade e a região geográfica. Esses achados subsidiam o planejamento regional específico de serviços de reabilitação protética e estratégias de saúde pública para reduzir as desigualdades em saúde bucal.

**Figure:**
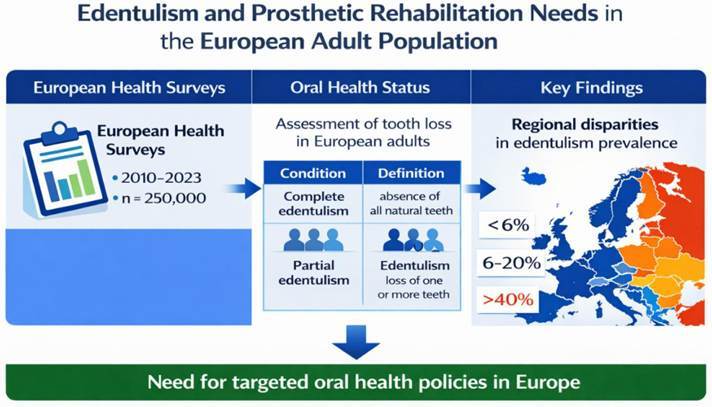

## Introduction

Tooth loss results from cumulative oral disease processes, primarily dental caries and periodontitis, and is associated with impaired mastication, reduced quality of life, and social disadvantage^1^ ^,^ ^2^ ^,^ ^3^. Despite advances in preventive dentistry, edentulism remains prevalent among older adults in Europe and continues to reflect inequalities in access to oral healthcare and preventive services.

National and European health surveys provide essential epidemiological data for monitoring oral health status and informing public health strategies. The European Health Interview Survey (EHIS) offers harmonised data that facilitate comparisons between countries^4^. However, access to prosthetic rehabilitation, particularly implant-supported treatments, varies substantially across Europe and is influenced by healthcare system organization, insurance coverage, and socioeconomic conditions^5^ ^,^ ^6^.

Although numerous studies have reported edentulism prevalence at the national level, comparative analyses across Europe remain limited by methodological heterogeneity, including differences in age group definitions, reliance on self-reported versus clinically assessed outcomes, and variability in survey design and sampling strategies. In addition, several available analyses rely on outdated data or focus on single countries, limiting their applicability to current European-wide planning.

The present study focuses on selected European countries for which recent and publicly accessible national survey data were available. These countries were selected to represent different geographical regions, healthcare systems, and economic contexts. The results do not represent all European countries but allow identification of major regional patterns in edentulism and prosthetic rehabilitation needs.

The objectives of this study were to determine the prevalence of complete and partial edentulism in selected European countries, to analyse demographic and geographical determinants of complete edentulism, and to describe implications for prosthetic rehabilitation at the population level.

## Material and methods

### Study Design and Data Sources

A cross-sectional ecological study was conducted using aggregated secondary data from publicly available European and national health surveys. Surveys published between 2010 and 2023 were included to reflect recent epidemiological trends. The study followed the STROBE guidelines for reporting observational research.

Data sources included the European Health Interview Survey (EHIS) waves 2 and 3, Eurobarometer 330 (Oral Health), and national oral health surveys from Germany^7^, Spain^8^, France^10^, Italy^11^, Poland^12^, Sweden, and the United Kingdom^9^. For countries without peer-reviewed survey publications, original data were obtained from official institutional websites and publicly accessible databases.

### Variables and Data Extraction

The primary outcome variable was complete edentulism, defined as the absence of all natural teeth. The secondary outcome variable was partial edentulism, defined as the loss of one or more natural teeth.

Independent variables included age group (35-44, 45-54, 55-64, 65-74, and ≥75 years), sex (male/female), and geographical region. Countries were grouped into Northern/Western Europe (Germany, Sweden, the United Kingdom), Southern Europe (Spain, France, Italy), and Eastern Europe (Poland, Romania). Extracted variables and their definitions are summarized in Table 1.

**Table 1:** Extracted variables, definitions, and categorization used in the analysis.

Variable category	Variable	Definition / description	Categories / coding
Demographic variables	Age group	Age at the time of survey, categorised according to standard groupings used in national and European health surveys	35-44 years; 45-54 years; 55-64 years; 65-74 years; ≥75 years
Demographic variables	Sex	Biological sex as reported in the original surveys	Male; Female
Geographical variables	Country	Country from which survey data were obtained	Germany, Spain; France, Italy; Poland, Romania, Sweden; United Kingdom
Geographical variables	Geographical region	Regional grouping based on geographical location and health system characteristics	Northern/Western Europe (Germany, Sweden, United Kingdom); Southern Europe (Spain, France, Italy); Eastern Europe (Poland, Romania)
Oral health outcomes	Complete edentulism	Absence of all natural teeth	Yes / No
Oral health outcomes	Partial edentulism	Loss of one or more natural teeth, with at least one remaining tooth present	Yes / No
Prosthetic rehabilitation	Removable prostheses	Use of removable dental prostheses, including complete dentures and removable partial dentures	Yes / No
Survey characteristics	Data source	Origin of the data used in the analysis	EHIS; Eurobarometer; National oral health surveys
Survey characteristics	Survey period	Year(s) of data collection or publication	2010-2023

### Statistical Analysis

Descriptive statistics were calculated as percentages and absolute numbers with corresponding 95% confidence intervals. Temporal trends were described when longitudinal data were available.

A generalised linear model with binomial distribution and logit link was fitted to estimate adjusted odds ratios for complete edentulism. Reference categories were age 45-54 years, Northern/Western Europe, and male sex. Multicollinearity was assessed using variance inflation factors. Statistical analyses were performed using R software version 4.3.1. Statistical significance was set at p < 0.05.

### Descriptive Analysis of Edentulism Prevalence

**Figure 1:**
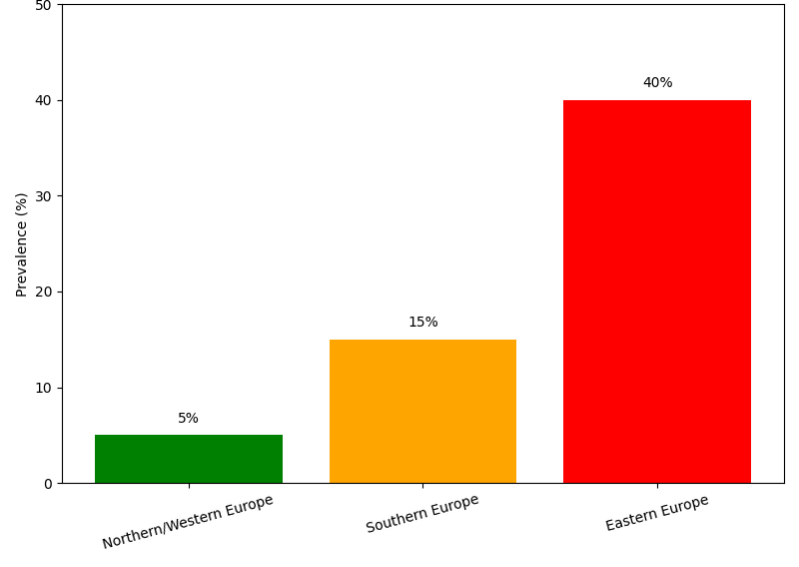
Prevalence of complete edentulism by European region (Age 65-74)

The prevalence of complete edentulism varied markedly across geographical regions (Figure 1). Among adults aged 65-74 years, prevalence was lowest in Northern and Western Europe, remaining below 6%, corresponding to approximately 3,200 individuals out of an estimated sample of 58,000. In Southern Europe, prevalence ranged between 6% and 20%, whereas in Eastern Europe it exceeded 40%, affecting approximately 4,500 individuals out of an estimated sample of 11,000.

**Figure 2:**
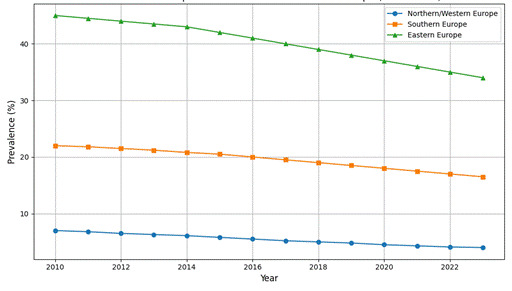
Evolution of complete edentulism prevalence in European (Age 65-74)

Temporal trends in complete edentulism from 2010 onwards are shown in Figure 2. A general decline in prevalence was observed across all regions over the study period; however, substantial regional differences persisted. Initial and final prevalence values for each region are indicated in the figure, and absolute numbers with 95% confidence intervals are reported in the text.

**Figure 3:**
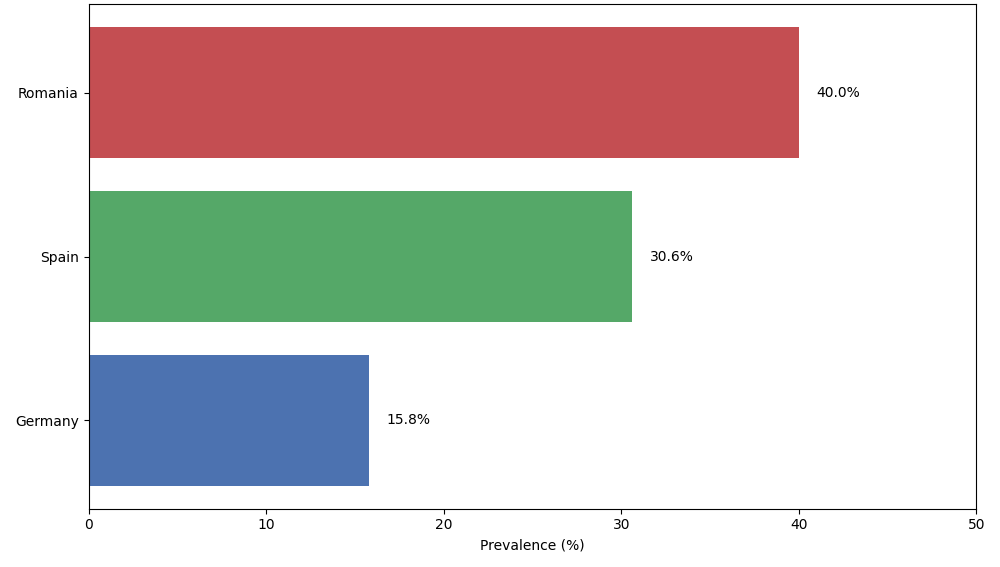
Prevalence of complete edentulism in Adults Aged 75+

Age-specific prevalence of complete edentulism increased progressively across all countries (Figure 3). In the ≥75-year age group, prevalence ranged from approximately 18% in Germany to over 55% in Romania, corresponding to absolute numbers exceeding 6,000 individuals in some national samples.

Partial edentulism was highly prevalent in all regions. Among adults aged 45-54 years, prevalence exceeded 65% in all geographical areas. In individuals aged 65-74 years, partial edentulism affected more than 90% of the population. Percentages and absolute numbers for partial edentulism by age group and region are reported in the Results section and in the original tables included in the manuscript.

Removable prosthetic rehabilitation included both complete dentures and removable partial dentures. Data on fixed partial dentures and implant-supported prostheses were inconsistently available across surveys and were therefore not included in the quantitative analyses. Regions with a higher prevalence of complete edentulism demonstrated greater reliance on removable prosthetic solutions.

### Multivariable Analysis

Multivariable modelling confirmed that age was the strongest independent predictor of complete edentulism. Compared with individuals aged 45-54 years, those aged 65-74 years had an adjusted odds ratio (aOR) of 11.2 (95% CI: 9.5-13.2), while those aged ≥75 years had an aOR of 28.4 (95% CI: 24.1-33.5).

The geographical region was also independently associated with complete edentulism. Relative to Northern and Western Europe, Southern Europe showed an aOR of 3.5 (95% CI: 2.9-4.2), and Eastern Europe an aOR of 9.8 (95% CI: 8.2-11.7). After adjustment, sex was not significantly associated with complete edentulism.

## Discussion

This study analysed the prevalence of complete and partial edentulism in selected European countries using aggregated data from national and European health surveys. Marked regional differences were observed, with a higher prevalence of complete edentulism in Eastern and Southern Europe compared with Northern and Western Europe. Age emerged as the principal determinant of complete edentulism across all countries.

The observed geographical gradient is consistent with previous reports linking oral health outcomes to socioeconomic conditions, healthcare system organisation, and access to preventive and restorative dental care^5^ ^,^ ^6^ ^,^ ^13^ ^,^ ^15^. Regions with higher prevalence of edentulism also demonstrated greater reliance on removable prosthetic rehabilitation, reflecting both treatment needs and limitations in access to fixed or implant-supported options^16^ ^,^ ^18^.

Comparison with data from non-European regions indicates similar patterns, with higher edentulism prevalence reported in settings characterised by lower socioeconomic indicators and reduced access to dental services^1^ ^,^ ^5^. These findings underline the relevance of demographic and structural factors in shaping oral health outcomes.

Several limitations should be acknowledged. The ecological design precludes individual-level inference, and heterogeneity among survey methodologies may affect comparability, particularly for partial edentulism, which is often self-reported. Differences in survey years, sampling strategies, and outcome definitions represent additional sources of variability. Despite these limitations, the consistency of findings across multiple independent data sources supports the robustness of the observed trends.

## Conclusion

The prevalence of edentulism in Europe shows substantial variation by age and geographic region. Advanced age and residence in Eastern and Southern Europe are associated with higher rates of complete tooth loss. These findings have implications for planning prosthetic rehabilitation services and for addressing persistent inequalities in oral health across Europe.

## Data Availability

The research data are available upon request.
